# Genotoxicity Studies Performed in the Ecuadorian Population

**DOI:** 10.1155/2012/598984

**Published:** 2012-02-16

**Authors:** César Paz-y-Miño, Nadia Cumbal, María Eugenia Sánchez

**Affiliations:** Instituto de Investigaciones Biomédicas, Facultad de Ciencias de la Salud, Universidad de las Américas, Ave. de los Granados y Colimes Quito, 1712842, Ecuador

## Abstract

Genotoxicity studies in Ecuador have been carried out during the past two decades. The focuses of the research were mainly the area of environmental issues, where the populations have been accidentally exposed to contaminants and the area of occupational exposure of individuals at the workplace. This paper includes studies carried out in the population of the Amazon region, a zone known for its rich biodiversity as well as for the ecological damage caused by oil spills and chemical sprayings whose consequences continue to be controversial. Additionally, we show the results of studies comprised of individuals occupationally exposed to toxic agents in two very different settings: flower plantation workers exposed to pesticide mixtures and X-ray exposure of hospital workers. The results from these studies confirm that genotoxicity studies can help evaluate current conditions and prevent further damage in the populations exposed to contaminants. As such, they are evidence of the need for biomonitoring employers at risk, stricter law enforcement regarding the use of pesticides, and increasingly conscientious oil extraction activities.

## 1. Introduction

Genotoxicity is a collective term that refers to any process that affects the structural integrity of DNA [1]. This multidisciplinary field of research aims to detect compounds capable of causing DNA damage in hopes of understanding the biological consequences of genotoxic agents and their involvement in the alteration of the molecular mechanisms of the genetic material [2]. These consequences can eventually lead to carcinogenic processes [3]. Over the past century, industrialization and globalization of the western hemisphere lead to the high volume production of different chemicals and complex preparations that are still currently released into the environment [4]. Living organisms are increasingly being exposed to genotoxic agents whose growing presence in the biosphere can substantially harm the population [5]. Activities such as fuel extraction and glyphosate spraying in the Amazon region of Ecuador are the two most controversial environmental health issues in the nation and are still considered as latent threats whose consequences continue to be studied [6, 7]. Agriculture in Ecuador is the second most important productive activity that contributes to national income [8]. However, the lack of regulation regarding pesticide use and occupational safety pose a significant threat to the workers' health [9]. Additionally, various studies have focused on individuals exposed to radiation in the workplace, such as medical radiation workers constantly exposed to ionizing radiation that has well-known DNA-damaging effects [10–12]. The present paper intends to show a summary on the work carried out in Ecuador for the past two decades in the field of genotoxicity. All the cytogenetic studies have been performed on blood lymphocytes and the results obtained refer only to somatic mutations. The studies have included cytogenetic findings, such as the ones presented in Table 1 as well as molecular results which are shown in Table 2.

## 2. Glyphosate Genotoxicity Studies

The northeastern Ecuadorian border underwent the aerial spraying of an herbicide mix during the period of 2002–2007 and was supported by the Colombian government [13]. The Roundup mix presumably contained high doses of glyphosate plus a surfactant known as polyethoxylated tallowamine (POEA) and the adjuvant Cosmoflux 411F [14]. Glyphosate is an effective organophosphorous herbicide used worldwide [15, 16] known to cause variable levels of toxicity in different organisms, such as the alteration of metabolic pathways, cytotoxicity in humans, metamorphosis alterations in amphibians, and abnormal development of sea urchin eggs [17–22]. Such reports add to the already numerous concerns over the compound's many effects in the environment. The cytogenetic study of blood lymphocytes from individuals that lived in the area that endured the sprayings showed the absence of chromosomal aberrations two years after the last spraying in Ecuadorian soil [13]. Also, since glyphosate has been known to cause oxidative stress in microorganisms, plant and animal species [23–29], the study analyzed three gene polymorphisms (*GSTP1* Ile105Val, *GPX-1* Pro198Leu, and *XRCC1* Arg399Gln) that have been previously associated to the alteration of antioxidant activity, DNA detoxicating processes, and protective functions [30–33]. The *GSTP1* gene encodes for glutathione S-transferase pi, an enzyme that is involved in the protection against exogenous and endogenous oxidative damage [34]. As a member of the gluthatione-S-transferase superfamily of enzymes, GSTpi participates in the conjugation of xenobiotics, such as herbicides, insecticides, and other environmental carcinogens, to form glutathione and facilitate their excretion [35, 36]. Specifically, the *GSTP1* Ile105Val polymorphism has been associated to higher levels of DNA damage in pesticide-exposed populations [37]. In the Ecuadorian population studied, the prevalence of the *GSTP1 *Val/Val genotype associated with enzyme dysfunction was observed in the exposed individuals. On the other hand, the *GPX-1* gene encodes for one of the most important detoxifying enzymes: gluthatione peroxidase. This enzyme protects mammalian cells, especially human erythrocytes, against oxidative damage [38]. Studies have shown that the loss of gluthatione peroxidase activity can generate tissue damage [39] and offer more sensitivity towards toxic xenobiotics, such as paraquat and adriamycin [38]. Although the *GPX-1* Pro198Leu polymorphism has been exhaustively studied in relation to cancer, the study in Ecuador identified the prevalence of the Leu allele of the *GPX-1* gene in glyphosate-exposed individuals that suggests a higher risk of DNA damage and increased sensitivity to herbicides. The third gene that was part of the study, *XRCC1*, is involved in the mechanisms of DNA single-strand breaks (SSBs) and base-excision repair (BER) that could modify the individual susceptibility to the genotoxic effect of xenobiotics [40, 41]. Although other studied populations have found an association between* XRCC1* gene genotypes and an increased risk of DNA damage due to pesticide exposure, similar results were not found for the Ecuadorian population studied [42]. Aside from the cytogenetic and molecular analysis carried out, the social conditions of the population were surveyed and psychological assessment was offered to all the individuals. The results of these two activities suggested the negative effect of the fumigations on the individuals' mental health, social conditions, and quality of life [13].

A previous study took place two years before the aforementioned study at the Ecuadorian border. It involved individuals living within 200 m to 3 Km from the areas under continuous and sporadic spraying [14, 43]. The comet assay technique, described by Singh et al., 1988 [44], was carried out on blood samples from exposed individuals and corresponding controls to show the occurrence of DNA fragmentation. DNA damage was classified into five categories and the mean of DNA migration was recorded. The results showed that the exposed group displayed significantly higher mean DNA migration than the control group. Similarly, there was a higher degree of DNA damage in the exposed group in comparison to the control group. These results suggest a negative effect of the glyphosate formulation since none of the studied individuals had been previously or simultaneously exposed to other toxic compounds, such as pesticides or tobacco [6]. The northern strip on the Ecuadorian border has not gone unnoticed in the controversy regarding aerial sprayings and their consequences. Nevertheless, the genotoxic and overall toxic potential of glyphosate remains under study and in vitro findings [45–49] have reached a variety of results [29]. The two studies carried out in the Ecuadorian border suggest a significant and immediate risk arising from the use of this chemical and prompt to continue its investigation.

## 3. Other Pesticide Genotoxicity Studies

Despite the known risks of the use of some pesticides due to their potential health consequences [25], many of those catalogued as extremely toxic continue being used in certain agricultural zones and flower plantations in Ecuador [9]. Pesticides are widely used all over the world in agriculture to protect crops and in public health to control diseases [50, 51]. The risk of developing malignancies such as cancer in occupationally exposed populations is of great concern and has drawn attention to workers in various activities, from the manufacturing workers to the pesticide applicators [52]. Studies available in scientific literature have focused their methodology on cytogenetic endpoints to evaluate the potential genotoxicity of pesticides, including chromosomal aberrations (CAs), micronuclei (MN), and sister chromatid exchanges (SCEs) [52, 53]. The analysis of chromosomal aberrations such as breaks, dicentric chromosomes, and rings was part of the methodology used in a leading pesticide exposure study carried out in flower plantation workers in Quito. These workers were exposed to 27 different pesticides, some of which have been previously labeled as highly toxic [9]. Also, the level of erythrocyte acetylcholinesterase was measures in every individual as a marker to evaluate the exposure to organophosphate pesticides [54]. The study found an overall CA percentage of 20.59% in the exposed group and 2.73% of CA in the control group. Additionally, the exposed group showed a higher proportion of chromatid-type aberrations and numeric alterations. This does not only reflect genomic instability but also comprises outstanding evidence of damage supported by the abnormal low levels of acetylcholinesterase seen in the exposed group [55].

Pesticide genotoxicity was also studied in individuals working as pesticide applicators in the zone of Cayambe, northeast from Quito. The workers were exposed to 46 pesticides of different degrees of toxicity and at different concentrations and mixtures during work at the plantation [56]. The methodology involved chromosomal aberration test matched with alkaline comet assay [55]. Additionally, the samples were analyzed at a molecular level focusing on the *CYP1A1* gene, a gene that has been extensively studied in relation to occupationally exposure to pesticides [56]. Because the gene is involved in the human xenobiotic metabolism, its alteration presumably increases the risk of developing lung, colorectal, prostate, and breast cancer [57–61]. In accordance to the first study carried out in workers from Quito, the results of the CA analysis in this study showed the significantly high presence of chromosomal damage in the exposed group as compared to the control group. Furthermore, the comet assay test offered results that supported the CA analysis by showing that the DNA migration of the exposed group was certainly higher than that of the control group. On the other hand, the study presented no correlation between the cytogenetic findings and genotyping of the *CYP1A1* MspI and Ile/Val gene polymorphisms. Though CA and comet assay showed interesting results, the gene was not linked to pesticide exposure in the studied population, as opposed to other populations [62, 63].

As an important element of the agricultural production, pesticides have become a necessary tool for crop management in developing countries [64, 65]. Nonetheless, the lack of adequate legislation and enforcement of existing pesticide laws and regulations places agricultural workers, their families, and nearby populations in great risk of developing cancer and other diseases [66]. Our study shows evidence of genotoxic damage in individuals occupationally exposed to pesticides in Ecuador. These are results that demand the establishment of effective exposure biomarkers that could be used for biomonitoring the threatened workers in order to prevent the future development of illness [5].

## 4. Hydrocarbons Genotoxicity Studies

The Ecuadorian Amazon is one of the ecologically richest regions in the world and it is also sparsely populated. The oil extraction activity in Ecuador began in 1972, it became economically fundamental immediately and continues to be the principal source of national income [67]. Unfortunately, along the process, millions of gallons of oil and toxic residues have been discarded directly onto the environment causing health and environmental issues [68–71]. Indeed, more than 30 billion gallons of toxic wastes and crude oil had been discharged into the land and waterways of the Ecuadorian Amazon up until 1993 [72]. Crude oil is a complex mixture of many chemical compounds. It contains a variety of hydrocarbons of diverse toxicological power such as benzene, toluene, xylene and polynuclear aromatic hydrocarbons [73]. High concentrations of benzene can cause neurotoxin symptoms that cause injuries to the bone marrow and, less frequently, pancytopenia [74]. Similarly, benzene is known to cause leukemia and the development of hematological tumors [75]. The exposure to carcinogen compounds used in the oil industry increases the development of cancer in men, women, and children. In men, an increase of lung, esophagus, rectum, skin, and kidney cancer has been noticed. In women, researchers have seen an increase of cervical, lymphatic ganglion, and bladder cancer. In children, an increase of hematopoietic cancer has been shown among other types of cancer [75–86]. Studies carried out in the Ecuadorian Amazon Basin were found to be compatible with international studies. A relationship between cancer incidence and living in proximity to oil fields has been established [87]. An initial study was carried out in the province of Orellana including 23 women living not more than 10 Km away from a crude oil extraction zone, with the corresponding control group. In order to assess genotoxicity, the comet assay test was used to measure DNA damage by classifying the nucleus morphology into five categories. The sampling zones closest to the extraction wells showed a greater evidence of DNA damage than those that are farther away which suggests a distance-damage relationship. This relationship is also supported by the increased occurrence of type A nuclei (no damage) as the distance from the wells increases [88]. Another study was carried out with a significantly bigger sample size from the nearby zone of San Carlos and matching controls from both San Carlos and the country's capital Quito. Comet assay showed that the affected group has a high occurrence of type B nuclei fragmentation, as opposed to the prevalence of type A cells in the control groups from San Carlos and Quito. Additionally, the analysis of chromosomal aberrations showed that 20% of the exposed individuals presented chromosomal breaks and gaps while only 2% of the control individuals had such aberrations [88]. At a molecular level, we incorporated the analysis of the polymorphisms of the genes *CYP1A1* (MspI and Ile/Val) and *MSH2 *(gIVS12-6T*>*C), both related to the development of cancer [58, 59, 89, 90]. The results of this last part of the study showed that the *CYP1A1* gene polymorphisms were not related to either group, as it has been reported previously in the Caucasian population [58]. However, the study showed a significant difference of the *MSH2 *gene polymorphism between groups which suggests a higher susceptibility to DNA damage in the exposed group [7]. The chemical complexity of petroleum causes that, once a spill occurs, the constituents disseminate into different extents between the oil phase and the air, soil and water phases of the environment. Physical, chemical, and biological processes age the spilled product resulting in additional changes in composition and complexity [91]. Taking in to consideration the toxicity of these fractions, the risk at petroleum extraction sites is an issue that must be addressed by making informed decisions. By comparing the affected group with the control individuals living in the same town though far from the extraction sites, the study has been able to evidence of the genotoxicity in the exposed population living in nearby petroleum extraction wells. This suggests that the contaminating material resulting from this activity has created an altered environment that exposes the population to chemical fractions considered as dangerous and may also cause genotoxic effects.

## 5. Radiation Genotoxicity Studies

Environmental mutagens can be broadly classified as radiation and chemicals [92]. Ionizing radiation is capable of extracting electrons of the radiated material due to its high energy. This is only a start point for other ionizing reactions that produce more unstable molecules that eventually cause mutations in DNA [93].

Ever since X-rays were shown to induce mutation in *Drosophila *over 70 years ago, the established idea has been that the genotoxic effects of ionizing radiation, such as mutations and carcinogenesis, are caused by the direct damage of the cell nucleus [94, 95]. Diagnostic radiology is a field of physical medicine that uses X-rays in order to obtain functional and anatomical information on the human body [96]. Because of the benefits of this diagnostic tool that allows real-time visualization, it is frequently used by the medical professionals [96].

Ionizing radiation is capable of acting on the living cell causing several effects that result from the excitation of atoms and molecules that ultimately cause structural changes. At a molecular level, DNA is possibly affected by water ionization that forms free radicals and promotes the oxidation of several compounds and hydrogen oxide [97]. Even small doses of this radiation could cause great damage because a simple electron excitation can break up to 20 hydrogen bonds [98–100]. The damage resulting from radiation exposure can be seen in the form of chromosomal aberrations in the cell nucleus that are associated with an elevating risk of developing cancer [101]. A first study focused on 10 individuals exposed to radiation in the workplace. They were exposed to 1.84 mSv/year and received a follow-up cytogenetic study after a year from the first blood sampling. The chromosomal aberration results found by the cytogenetic analysis showed interesting results in both instances [102]. First, they showed that complex chromosome alterations, such as rings and dicentrics, are present in low percentages contrary to the occurrence of simple alterations (gaps, breaks, and acentrics). This is due to the fact that 72 h cultures were used. By then, cells have gone through a second and third mitotic divisions. Therefore, primary alterations have been kept and turned into secondary aberrations in the growing generations; meanwhile, early cells with complex alterations have already died [103, 104]. Two individuals exposed to higher doses of radiation (4.54 and 1.07 mSv, resp.) did show complex alterations in the second sampling. This is an unusual finding likely to be caused by the individuals' sporadic exposure to higher amounts of radiation. A significant increase of chromosomal aberrations was observed by comparing CA during the first and the second sampling. Out of these, there was a higher number of lesions at the chromatid level (mostly gaps) possibly due to the proper action of DNA repair mechanisms at low doses of exposure over long periods of time [102, 105]. Other studies have also found an increase of CA in individuals exposed to similar doses of radiation, but have not addressed the importance of periodic biomonitoring [106]. Though numerical aberrations were not the focus of the study, the exposed individuals showed an increased frequency of hyperploidies and hypoploidies possibly due to the imbalance in the cell cycle caused by the exposure to toxic agents [107]. This data evidences the importance of periodic control of the occupational exposure and of monitoring the dosage-time levels of exposure at the workplace [102].

A second study focused on telomeric associations in individuals exposed both to X-rays and smoke in order to determine the existence of these associations as chromosomal markers of exposure to these carcinogenic agents. The phenomenon of telomeric association is an intermediate step in the progression towards chromosomal instability that also comprises a risk of developing cancer [108, 109]. Cytogenetic monitoring is currently accepted as an evaluation tool for exposed populations at risk as it has been used in studies regarding ionizing radiation [5]. Cytogenetic analysis of cigarette smokers has shown the occurrence of chromosomal aberrations in populations from Colombia and India [110, 111]. In this study, mitotic indices determined in all groups (smokers exposed to radiation, nonsmokers exposed to radiation, smokers unexposed, and unexposed nonsmokers) showed no correlations between the exposure to both carcinogens and the mitotic indices and cell proliferation. Nonetheless, the three different exposed groups showed high frequencies of telomeric associations [112]. These results were particularly surprising for cigarette smokers since no cytogenetic biomarker of exposure for cigarette had been demonstrated to be consistent so far. Also, the group of smokers unexposed to radiation showed higher frequencies of TA than the nonsmoking X-ray-exposed group, a result that was possibly due to the many carcinogens present in cigarette smoke [110, 111, 113]. Though it has been reported that both agents have a synergistic effect [113], our study did not find such a tendency. However, the group exposed to both agents did show the highest frequency of telomeric associations. The study suggested that telomeric associations can assess the genomic instability phenomena in populations exposed to mutagens. Although, telomere length has been reported as a biomarker for age, stress and cancer, telomere biology and the molecular pathways that protect telomeres continue to be studied in order to determining the outcome of radiation exposure [114].

In another study, the inclusion of gaps as chromosomal aberration was investigated. Gaps are defined as the unstained regions of a chromosome that contain zones of lesser width than that of a chromatid [115, 116]. A gap is observed as an empty space because the DNA thread is so thin that it becomes practically invisible to the usual technique [113]. Since genotoxic agents such as ionizing radiation are capable of inducing chromosomal uncoiling events and affecting DNA condensation, gaps can certainly be the product of exposure to genotoxic agents [117]. Nonetheless, some authors had considered gaps to be structures that lack biological significance [118]. The study involved individuals exposed to X-rays and the unexposed control group. The findings of the CA analysis and comet assay were compared, including and excluding gaps. These two complementary techniques do not detect the same kind of lesions. On one hand, chromosomal aberrations are originated from double-strand breaks; on the other hand, comet assay can detect single-strand breaks, double-strand breaks, and alkali labile sites (when using the alkaline version) [119] and has proven to be a useful way of assessing X-ray damage to lymphocytes [120]. The correlation between the two methods including gaps as CA was positive. Gaps measured damage in the DNA since there was a stronger correlation between the results of both applied techniques when gaps were included as a CA. Although there is an increasing interest in studying the more complex chromosomal aberrations such as dicentrics and translocations, current studies still include gaps as part of the genotoxicity studies [121–124]. These findings suggested a revision of the biological importance of gaps in population occupational biomonitoring [124]. Furthermore, another study involved a group of radiologists and technicians exposed to X-rays at the workplace, excluding those with family and personal history of cancer and smoke exposure. The mean dose of ionizing radiation for the affected group was 0.99 mSv and the chromosomal aberrations observed involved gaps, breaks, dicentric, rings, and double minutes. The cytogenetic analysis showed that CAs were present in 50% of the individuals in the exposed group and in 2% of the control individuals. However, these results were not statistically significant. On the other hand, the comet assay did show a highly significant difference of migration in the exposed group as compared to the control group, possibly because the comet assay shows a wider set of damage consequences [93, 125]. Similarl to a study carried out in Iran that found no specific relation with the characteristics of the occupational setting and the duration of exposure [126], this study found no correlation between the results of both tests and the duration and dose of exposure due to a lack of significant variation between individual doses. Such results may also support the idea of hormesis taking place as a way of adapting to the workplace after several years, though the idea remains to be controversial [127]. Even though there were no significant results regarding CA, relative risk calculation showed that exposed individuals had a risk 20 times higher of showing aberrations than the control group [128]. These aberrations may lead to the alteration of cell control mechanisms such as apoptosis and tumor suppressor genes, besides the loss of genetic material due to cell death as a result of changes in division and repairing mechanisms [129–131]. Although this study did not show an association between CA and exposure time or dose level, other studies have agreed on the fact that long-term exposure to low radiation levels are the cause for higher percentages of CA [132, 133].

Cytogenetic findings are of great importance because they are associated with the mechanisms of carcinogenesis. The interaction with physical agents, such as ionizing radiation, produces a variety of primary lesions [134] whose prevalence can determine cancer risk [135]. Because of the importance of biomonitoring occupationally exposed populations, proper research guidelines have been established [136]. mFISH assays are currently being put in use in order to carry out a more detailed analysis of simple and complex aberrations that could model the effects of radiation on lymphocytes [107]. Nonetheless, earlier cytogenetic techniques are still held as the golden standard for biomonitoring populations. Environmental and occupational health issues are increasingly being studied because of its importance in public health. In Ecuador, going through a preliminary cytogenetic testing to evaluate the genotoxic effects of different agents is a rather voluntary decision and the toxic qualities of certain widely used chemicals, such as herbicides and pesticides, are not of common knowledge. According to the results obtained in this set of studies, adequate biomonitoring laws should be enforced. In the case of glyphosate, research has helped to consider changes regarding the targeted areas, duration of sprayings and chemical composition of herbicides. Though plantations and industries do offer protective equipment, this gear is not always used by the small farm owner. Reports on the use of highly toxic pesticides conclude that the lack of regulation of pesticide use benefits the informal distribution of these hazardous compounds, not only in flower plantations but increasingly in small farms. Lastly, occupational health risks must be studied in all professions that face any level of exposure to physical or chemical agents suspected to cause illness. Radiologists and other professionals exposed to radiation should have access to cytogenetics testing and follow-up studies that can report on any unusual results in order to prevent diseases as part of occupational health and safety laws.

## Figures and Tables

**Table 1:** Cytogenetic findings in genotoxicity studies.

Glyphosate	Paz-y-Miño et al. [6]	Comet assay: 35.5 *μ*m DNA migration for exposed, 25.94 *μ*m for controls.
	Paz-y-Miño et al. [13]	All the studied population showed low or no chromosomal fragility.

Other pesticides	Paz-y-Miño et al. [9, 10, 93]	Chromosomal aberrations: 20.59% in exposed and 2.73% in controls.
	Paz-y-Miño et al. [55]	Comet assay: 31.58 *μ*m DNA migration for exposed, 25.94 *μ*m for controls. Chromosomal aberrations: 5.48% in exposed and 0.45% in controls.

Hydrocarbons	Paz-y-Miño et al. [7]	Chromosomal aberrations: 20% in exposed and 1-2% in controls. 12% type A DNA damage and 1% type E DNA damage in exposed group while 81% type A and 0% type E in controls.
	Paz-y-Miño et al. [88]	48.8% type A DNA damage and 0.1% type E DNA damage in exposed group while 67.9% type A and 0% type E in controls.

Radiation	Paz-y-Miño et al. [102]	Chromosomal aberrations: 29% in exposed, 26.0% in the followup, and 3.5% in controls.
	Paz-y-Miño et al. [112]	12.6% metaphases with telomeric associations in the exposed smoker group; 6.0% TA in exposed nonsmokers; 9.0% in unexposed smokers and 0.1% TA in control group.
	Paz-y-Miño et al. [9, 10, 93]	Comet assay: 26.55 *μ*m DNA migration; mean chromosomal aberrations without gaps: 5.39% (*r* = 0.50, *P* < 0.05). Mean chromosomal aberrations including gaps: 12.08% (*r* = 0.78, *P* < 0.01).
	Muñoz et al. [128]	Comet assay: 29.08 *μ*m DNA migration for exposed group, 25.91 *μ*m for controls. Chromosomal aberrations: 50% in exposed, 26.0% in the followup, and 4% in controls.

**Table 2:** Molecular findings in genotoxicity studies.

Glyphosate	Paz-y-Miño et al. [13]	Regarding the *GSTP1* Ile105Val polymorphism, the frequency of the Val allele was higher in exposed individuals (0.48) than control individuals (0.28). The Val/Val variant represented a 4.88-fold risk of acquiring detoxification problems, whereas the combination of the Ile/Val and Val/Val alleles was associated with a 2.6-fold risk of presenting a *GSTP1* gene dysfunction. As for the *GPX-1* Pro198Leu polymorphism, the Leu allele had a higher frequency in exposed individuals (0.41), unlike control individuals (0.32). The Leu/Leu variant was associated with an 8.5-fold risk of having problems in the function of the *GPX-1* gene.

Other pesticides	Paz-y-Miño et al. [55]	The level of damage was not significantly influenced by genetic polymorphisms of the *CYP 1A1* gene in the studied population.

Hydrocarbons	Paz-y-Miño et al. [7]	As far as the *MSH2* gene is concerned, there is a relation between polymorphisms of the exon 13 and the DNA damage evaluated in the individuals exposed to hydrocarbons (*P* < 0.001), the study of the *CYP 1A1* gene found no relation between its polymorphisms and having greater susceptibility to DNA damage.
